# Supplementary material for: The relationship between home ownership and fall-related outcomes: The National Health and Aging Trends Study
Source: PLOS Glob Public Health. 2021 Dec 22;1(12):e0000019. doi: 10.1371/journal.pgph.0000019 (PMC10021867; doi:10.1371/journal.pgph.0000019)
Supplement: Attachment — Submitted filename: Response to Reviewers.docx [file pgph.0000019.s001.docx]

- *Ching-Yao Tsai, Tao-Hsin Tung, Yang-Tzu Li, Wei-Cheng Chen , “The relationship between home ownership and fall-related outcomes: The National Health and Aging Trends Study” (Manuscript Number: PGPH-D-21-00155)*

Dear Editor,

Thank you for your 21^th^ June, 2021 letter with the referee comments. We had copyedited the manuscript based on the suggestions from Editage. We thank you for the reviewer and the editor for useful suggestions. We hope the correction of the revised manuscript is satisfactory and met the requirement of *Plos one*. We are happy to make any further change if required

Dear Author,

Thank you for choosing to work with Editage!

Please go through the changes and comments in the edited file.

If you have questions about the edit, have received journal comments, or would like us to check additional revisions made by you, please use the **Post Order Assistance** option on your EditageOnline^TM^ account (<http://app.editage.com/>).

We’d also like to know what you thought of our work and how we can do better. Please send us your feedback on the assignment through your EditageOnline^TM^ account.

**Acknowledging editing support**

Several authors choose to acknowledge Editage’s editorial support in their paper. According to prominent publication guidelines such as the ICMJE guidelines on authorship, editing or writing support should be acknowledged in the paper. Such acknowledgments also serve to assure journal editors/reviewers that the English has been thoroughly reviewed, and meets the required standards for publication.

If you would like to acknowledge our editorial support for this paper, you can do so by including the following sentence in the Acknowledgments section of your paper:

*We would like to thank Editage (www.editage.com) for English language editing.*

Regards,

Your editor

**Additional notes**

**Formatting**

(To ensure that your paper conforms to the formatting requirements of the target publication)

1. Short title: Limit to 100 characters
2. Author list: Enter author names on the title page of the manuscript and in the online submission system. On the title page, write author names in the following order: First name (or initials, if used), Middle name (or initials, if used), Last name (surname, family name). Each author on the list must have an affiliation. The affiliation includes department, university, or organizational affiliation and its location, including city, state/province (if applicable), and country. Authors have the option to include a current address in addition to the address of their affiliation at the time of the study. The current address should be listed in the byline and clearly labeled “current address.” At a minimum, the address must include the author’s current institution, city, and country. If an author has multiple affiliations, enter all affiliations on the title page only. In the submission system, enter only the preferred or primary affiliation. Author affiliations will be listed in the typeset PDF article in the same order that authors are listed in the submission.
3. Corresponding author: The submitting author is automatically designated as the corresponding author in the submission system. The corresponding author is the primary contact for the journal office and the only author able to view or change the manuscript while it is under editorial consideration. Only one corresponding author can be designated in the submission system, but this does not restrict the number of corresponding authors that may be listed on the article in the event of publication. Whoever is designated as a corresponding author on the title page of the manuscript file will be listed as such upon publication. Include an email address for each corresponding author listed on the title page of the manuscript.
4. Author contribution: Provide at minimum one contribution for each author in the submission system. Use the CRediT taxonomy to describe each contribution
5. Acknowledgements: Those who contributed to the work but do not meet our authorship criteria should be listed in the Acknowledgments with a description of the contribution.
6. References: Use Vancouver style in creating your references.

**Editor’s report**

I have provided feedback on your manuscript through specific comments along with ratings for relevant sections. The key below the table explains my ratings. I hope you find my feedback useful.

| Section | Rating |
| --- | --- |
| **Title**  An effective title is concise while being representative. | ★★★ |
| **Abstract**  A good abstract explains the aims of the research, how these were met, and the main findings. | ★★★ |
| **Introduction**  This section should set the context for the study, clearly state the research objective, and establish the significance of the study. | ★★★ |
| **Materials and methods**  This section should completely describe all methods, techniques, and instruments used. This includes ethical considerations. | ★★★ |
| **Results and discussion**  These sections should present the data and findings in a clear and unbiased manner, and address the objective or research question stated in the introduction. | ★★★ |
| **Conclusions**  A good concluding section notes the limitations of the study. It should mention the scope for further research as well as the implications/application of the study. | ★★★ |

★★★	This section required only a few revisions.

★★	Most parts of this section required revision.

★	The entire section required significant revision. Please go through my comments/changes carefully.

| **Comments** | |  |
| --- | --- | --- |
|  | I have made changes to improve clarity and readability in the document so as to improve its impact. In case any of these changes do not convey what you wish to say, then please do get back to me with a clarification. I would encourage you to get back to me for another round of edit after you have accepted/rejected my changes, addressed the comments, and made any further revisions, in order to obtain a more polished document for submission. Kindly take note of the missing segments of the paper, and have these ready if you decide to let us go through your manuscript before submission. Please contact the Client Manager for details so that we can continue to work together to make this manuscript submission ready.  **SCOPE**  Your target journal, *PLoS One*, is an inclusive journal community working together to advance science for the benefit of society, now and in the future. Founded with the aim of accelerating the pace of scientific advancement and demonstrating its value, we believe all rigorous science needs to be published and discoverable, widely disseminated and freely accessible to all. The research we publish is multidisciplinary and, often, interdisciplinary. PLOS ONE accepts research in over two hundred subject areas across science, engineering, medicine, and the related social sciences and humanities. We evaluate submitted manuscripts on the basis of methodological rigor and high ethical standards, regardless of perceived novelty. As the journal comprises the field of research relevant to your study topic, I believe that your manuscript will fit well in that field.  **NOVELTY OF THE STUDY**  The novelty of this study needs to be further highlighted. This may be done by stating where this report is in terms of the current research on this topic. This is important so that readers know where your article stands.  **RELEVANCE AND CONTRIBUTION OF THE STUDY**  The relevance of the study may be further highlighted by stating clearly how this can help clinicians in identifying, diagnosing, managing, and treating patients with similar diseases. Consider adding this to your abstract and or manuscript.  **SUBMISSION READINESS**  Overall, a good number of changes were made throughout the manuscript. Given the revisions that I have done, I recommend that this paper should go through another round of editing before the author submits the final manuscript to include the missing segments of the paper. Please go through my revisions and comments and edit accordingly.   - The title is concise and avoids unconventional abbreviations as required by the journal guidelines. It will be able to draw the attention of potential readers to your abstract. There is no need for a major change. - The Abstract was good and set up the primary motivation for presenting this study, given its potential implications. The methods and major findings were succinctly described, and a concluding statement giving the implications of the findings was also present. Changes were made to improve readability. My only comment to expound more on the importance of doing this study and how further studies can be stemmed from it. - The Introduction provided sufficient background to help readers evaluate your work. You have clearly stated the purpose of the study. Moreover, the transition between sentences and paragraphs was good. A few grammatical and language changes were made. - The Methods were sufficiently detailed to permit a clear understanding and replication of the study’s methodology. A clear logical order was followed and a separate section on the statistical analyses was provided. - The Results section is primarily devoted to the description of the study findings and makes exemplary use of statistical analysis to scientifically validate the findings. Data interpretation and comparison with other sources, e.g., other data from your study or data taken from the literature, should only take place in the Discussion. You clearly adhered to this rule of data presentation. The narrative is well structured by your subheadings. The Results highlighted all the major outcomes of your study. - The Discussion section is well organized, as it begins with a summary of the main findings, followed by juxtaposing such findings with the current literature. This is mainly a well-presented section discussing all relevant study findings. Your narrative never loses focus and develops with an inherent logic. Many languages changes were made in this section to avoid wordy sentence that affected readability. Please highlight further the importance and relevance of the study and its implications on the current body of knowledge. | |

**Quick tip**

**GUIDELINE**

Redundancy, that is, the unnecessary repetition of words or ideas, should be avoided in academic writing.

**EXPLANATION**

In academic writing, ideas need to be conveyed as concisely as possible and redundant expressions should be avoided. For example, it is redundant to say “The boy was bored throughout the entire play.” Here, the word “throughout” means during the whole time or period and already implies “entire.” Redundancy can be avoided in this sentence by removing the word “entire.”

Correct sentence: “The boy was bored throughout the play.”

**Example**

**Original sentence:** “However, the reason why FOF was more common among females still needs needed researchers to focus on this issue in the future further research.”

**Revised sentence:** “However, the reason why FOF was more common among females still needs further research.”
