# Supplementary material for: The relationship between home ownership and fall-related outcomes: The National Health and Aging Trends Study
Source: PLOS Glob Public Health. 2021 Dec 22;1(12):e0000019. doi: 10.1371/journal.pgph.0000019 (PMC10021867; doi:10.1371/journal.pgph.0000019)
Supplement: Attachment — Submitted filename: Response to Reviewers4.docx [file pgph.0000019.s002.docx]

- *Ching-Yao Tsai, Tao-Hsin Tung, Yang-Tzu Li, Wei-Cheng Chen , “The relationship between home ownership and fall-related outcomes: The National Health and Aging Trends Study” (Manuscript Number: PGPH-D-21-00155)*

Dear Editor,

Thank you for your 6^th^ October, 2021 letter with the referee comments. We thank the reviewer and the editor for useful comments and suggestions. We have substantially revised the paper as requested. The important issues raised by the reviewer have been clarified, corrected, and elaborated. We hope the correction of the revised manuscript is satisfactory and met the requirement of *Plos One*. Please find the revised manuscript and an outline of reply to the referee. We are happy to make any further change if required.

1. The 'Competing Interests' statement has been added in Page 14, Line 13. in 'Revised Manuscript with Track Changes'.

2. The Financial Disclosure statement has been added in Page 14, Line 15 in 'Revised Manuscript with Track Changes'.
